# Antifungal activity of *Lysinibacillus macroides* against toxigenic *Aspergillus flavus* and *Fusarium proliferatum* and analysis of its mycotoxin minimization potential

**DOI:** 10.1186/s12866-023-03007-4

**Published:** 2023-09-26

**Authors:** Ahmed Lotfy E. Mahmoud, Ayat H. A. Mohamed Kilany, Elhagag A. Hassan

**Affiliations:** Botany and Microbiology Department, Faculty of Science, Assiut University, Assiut, 71516 Egypt

**Keywords:** Biological control, Chitinase activity, Fumonisin B_1_, Mycotoxins, *Lysinobacillus macroides*

## Abstract

**Background:**

Toxigenic fungi (*Aspergillus* and *Fusarium*) and their metabolites represent the major cause of corn and corn-based products contamination and consequently lead to severe economic and health issues.

**Aim:**

Our current study aimed to investigate the efficacy of using *L. macroides* Bac6 as a biological control agent against the toxigenic fungi; *A. flavus* f10 and *F. proliferatum* f30 and their mycotoxins.

**Results:**

The results illustrated that *A. flavus* f10 produced the aflatoxins AFB_1_ and AFG_2_ with concentrations of 21.239 and 13.593 ppb, respectively. While *F. proliferatum* f30 produced fumonisin B_1_ (9600 ppb). Furthermore, *L. macroides* showed a high potential for inhibition of toxigenic fungal growth using a dual culture method. *F. proliferatum* f30 and *A. flavus* f10 were found to be inhibited by a percentage of 80 and 62.5%, respectively. The results were confirmed using the scanning electron microscope. The antagonistic bacteria, *L. macroides*, showed chitinase productivity and activity of 26.45 U/L and 0.12 U/mL/min, respectively, which illustrates its potential application as a biocontrol agent. The GC-MS analysis revealed an abundance of Pyrrolo[1,2-a] pyrazine-1,4-dione, Hexahydro in the bacterial supernatant that exhibited antifungal characteristics. *L. macroides* had a significant reduction of AFB_1_ and AFG_2_ produced by *A. flavus* f10, recording 99.25% and 99% inhibition, respectively. It also showed strong inhibition of fumonisin B_1_ (90% inhibition) produced by *F. proliferatum* f30. Conclusion: Thus, the current study is a prospective study evaluating for the first time the potential impact of *L. macroides* *Bac6* against the toxigenic fungi and their toxins.

**Supplementary Information:**

The online version contains supplementary material available at 10.1186/s12866-023-03007-4.

## Background

The world’s population is estimated to be 8 billion by 2025 and 9.8 billion by 2050 [1]. As a result, increasing global agricultural productivity is required to meet food demand [2, 3]. Grains, pulses, and oil seeds continue to play an important part in both human and animal nutrition around the world. *Aspergillus flavus* is typically found in decaying vegetation and soil [4]. This fungus is well-known for producing aflatoxin, a strong carcinogen that is dangerous to both people and animals [5, 6]. *A. flavus* may infect a diverse range of hosts, such as maize, cotton, and peanuts. [7]. Infections that develop during growth or storage might cause significant monetary losses [8]. Due to its capacity to create secondary metabolites such as aflatoxins, cyclopiazonic acid, and kojic acid, *A. flavus* is fatal [9]. In the host organism, these metabolites may result in cell damage and death [10]. *A. flavus*’s aflatoxins have the potential to harm people either immediately after exposure or over time, leading to conditions such as liver cancer, immune system suppression, and growth retardation [11, 12]. A filamentous fungus called *Fusarium proliferatum* infects maize and causes illnesses in plants. It creates mycotoxins, which, when consumed by people or animals eating tainted feed or crops, can have major negative effects on their health [13]. *F. proliferatum* infects maize plants, causing root, stalk, and ear rot as well as producing fumonisins, a mycotoxin that can taint maize kernels and pose a major risk to human and animal health, results in a serious health risk to consumers. [14]. Additionally, grains, including corn (maize), are susceptible to mycotoxins before and after harvest [15]. When specific fungal species are exposed to environmental conditions (e.g., water activity, temperature, pH, and intergranular gas composition), mycotoxins are produced. Mycotoxins from toxigenic fungal growth are produced in food and animal feeds via the secondary metabolism process [16]. Mycotoxins, in contrast to many bacterial toxins, are typically extremely stable when exposed to the conventional heating method used in the preparation of foods for human consumption [17]. These fungi and mycotoxins have serious consequences for human and animal growth and health [18–20]. Aflatoxin and fumonisin B_1_ are two mycotoxins that have been linked to cancer in humans and other mammals as well as renal and neurological disorders. [21, 22]. One of the primary issues in managing mycotoxigenic fungi and related mycotoxin, which is a source of contamination is that fungicides are ineffective at treating agricultural commodities, that are usually applied in the agricultural sector to treat diseases caused by fungi. [23, 24]. Biological control may be a long-term solution, safe for human and environmentally healthy, self-sustaining treatment strategy to manage mycotoxins, resulting in reduced agri-operational costs [25, 26]. Biological control is effective, and many new approaches are being developed, most of them are based on microbiological research and the usage of microbial organisms that inhibit fungal growth and detoxify mycotoxins [27]. Bio-acceptable approaches should not only be beneficial to the environment and the crop, but also to the producers and the consumers [27]. One of the approaches that are most important for ensuring humanity’s health and sustaining eco-friendly food production will unquestionably be the introduction and administration of biological and natural protective agents against fungal contamination. [28, 29]. Biocontrol methods include an antibiosis, mycoparasitism, competition, development of resistance in the host plant, and competition [30]. As a result, mycotoxigenic biocontrol by antagonistic agents is regarded as a promising strategy for minimizing and reducing toxin production by these fungal species, and thus reducing the hazards of these toxins on human health when consuming food products [31]. *Flavobacterium aurantiacum B-184* had been effectively evaluated for aflatoxins destruction and was effective in eradicating *Aspergillus* toxins from liquids irreversibly [32, 33]. *Lactobacillus plantarum CECT 749 CFS* had a strong antifungal impact on maize kernels and maize ears against *A. flavus* and *F. verticillioides*, and FB1 and AFB1 levels were extremely dropped [34]. *Bacillus velezensis* RC 218 and *Streptomyces albidoflavus* RC 87B successfully reduced *Fusarium* Head Blight by up to 30%, its severity by up to 25%, and deoxynivalenol accumulation by up to 51% on durum wheat under field conditions [35, 36]. Interestingly, *Lysinibacillus macroides*, isolated from damaged Waste materials from fruits and vegetables were found to possess an inhibiting effect on food-borne microbes [37]. It has been proven that sugarcane bagasse can be applied for producing extracellular laccase on a large scale using *Lysinibacillus macroides* at a low cost and with ease [38]. Additionally, *Lysinibacillus macroides* produces some antimicrobial compounds and chitinase, glucanase, and protease, which are enzymes that have the ability to degrade the cell wall, are well-known to be used as biocontrol techniques. [39]. *Lysinibacillus* has the ability to down regulate the fungal-hyphal growth [40]. Additionally, by lowering the prevalence of *Salmonella*, which is well-known for producing significant morbidity in poultry, people, and other animals such as cattle and pigs, as well as in several plants, *Lysinibacillus macroides* was working as a natural control agent [41]. The most common way for these illnesses to spread is through the intake of contaminated food and drink [41]. It is proven that *Lysinibacillus macroides* shows a significant concentration reduction ability for chromium (VI), which is known as an accumulated pollutant in lakes in Mexico [42]. *Lysinibacillus has been proven to be effective at pest management* [43], improve crop production and clean up heavy metal-contaminated ecosystems [43]. Zinc-tolerant *Lysinibacillus spp.* are additionally known to boost maize production in Zinc-contaminated soil [43, 44].

All across the world, mycotoxins pose a serious threat to the safety of food. Farmers and consumers suffer financial losses in poor nations such as Egypt when food lots contain high toxin levels. Extremely polluted lots are either totally discarded or sold for an undesirable price. In years and locations with extremely high levels of mycotoxins, leading to significant food loss and waste. In low- and middle-income countries, where mycotoxin limits may not even exist or may not be routinely implemented, human health consequences can be substantially get worse [5]. For the bio-management of toxic fungus and their potential to manufacture mycotoxins, the use of bacterial strains is recommended as a non-chemical, successful, environmentally friendly, and affordable biological control strategy [45]. There are no published papers that demonstrate the potential effect of using *L. macroides* as a natural deterrent towards toxigenic fungi that are related to corn and its products; therefore, we aim for the first time to investigate the efficacy of *L. macroides* Bac6 as a natural barrier to toxigenic fungi (*A. flavus* f10 and *F. proliferatum* f30) and minimize their mycotoxins to reduce the loss of crops and increase the productivity and quality of the corn and its products, which will reflect on the consumers’ health.

## Results

### **Molecular studies for the most predominant toxigenic fungi isolated from yellow-corn and cornflakes (*A. flavus*f10 and*F. proliferatum*f30) strains**

From the previous screening study performed by [46], *A. flavus* f10 and *F. proliferatum* f30 showed the highest productivity of mycotoxins, so they are representative producers of aflatoxins and fumonisin B_1_, respectively. *A. flavus* f10 and *F. proliferatum* f30 isolates were identified at the species level using macro- and microscopic characteristics. Moreover, *A. flavus* f10 and *F. proliferatum* f30 isolates were subjected to confirmation of their identification using molecular techniques. The molecular typing resulted in partial 18 S rRNA gene sequences of 582 and 521 bp for *A. flavus* f10 and *F. proliferatum* f30, respectively. The partial 18 S rRNA gene sequence of 582 bp of the representative *A. flavus* f10 isolate had a sequence with 99% similarity to *Aspergillus flavus* strain PUXX-FS06 (KR296888.1), *Aspergillus flavus* IFM 42,126 (LC602022.1), *Aspergillus flavus* strain TN533D12 (MH271095) and *Aspergillus flavus* strain USMG09 (KF434090.1) available in the GenBank database (S 1 A). The candidate isolate was identified as *Aspergillus flavus* f10, which belongs to the family Aspergillaceae, order Eurotiales, class Eurotiomycetes. Similarly, the partial 18 S rRNA gene sequence of 521 bp of *F. proliferatum* f30 isolate had a sequence with 100% similarity to *Fusarium proliferatum* strain HC01-1 (MT560215.1), *Fusarium proliferatum* strain TH12-5 (MT560218.1) and *Fusarium proliferatum* strain CF2 (MN658457.1), which are available in the Genbank database (S 1B). The chosen isolate was identified as *Fusarium proliferatum*, which belongs to the family Nectriaceae, order Hypocreales, class Sordariomycetes. The phylogenetic trees were built independently from various sequence alignments of 18 S rRNA genetic sequences. The obtained sequences of toxigenic fungal strains *A. flavus* f10 and *F. proliferatum* f30 were deposited in the genebank database under accession numbers OQ087136 and OQ087105, respectively.

### **Quantification of mycotoxins production by*A***. ***flavus*f10 and*F***. ***proliferatum*f30 strains using the HPLC technique**

**Fig. 1:**
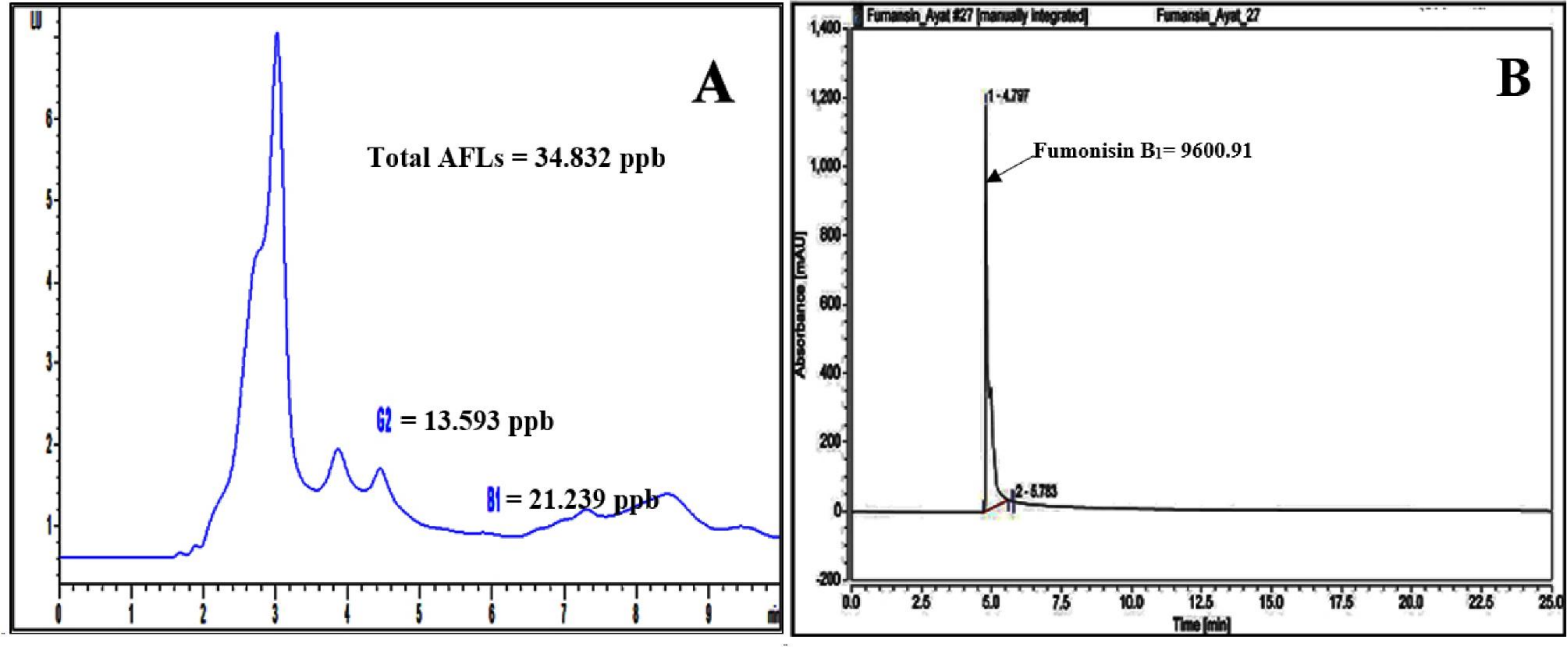
The chromatograms of the recovered mycotoxins which produced by the toxigenic **(A)***A. flavus* f10 and **(B)***F. proliferatum* f30 strains

**Fig. 2:**
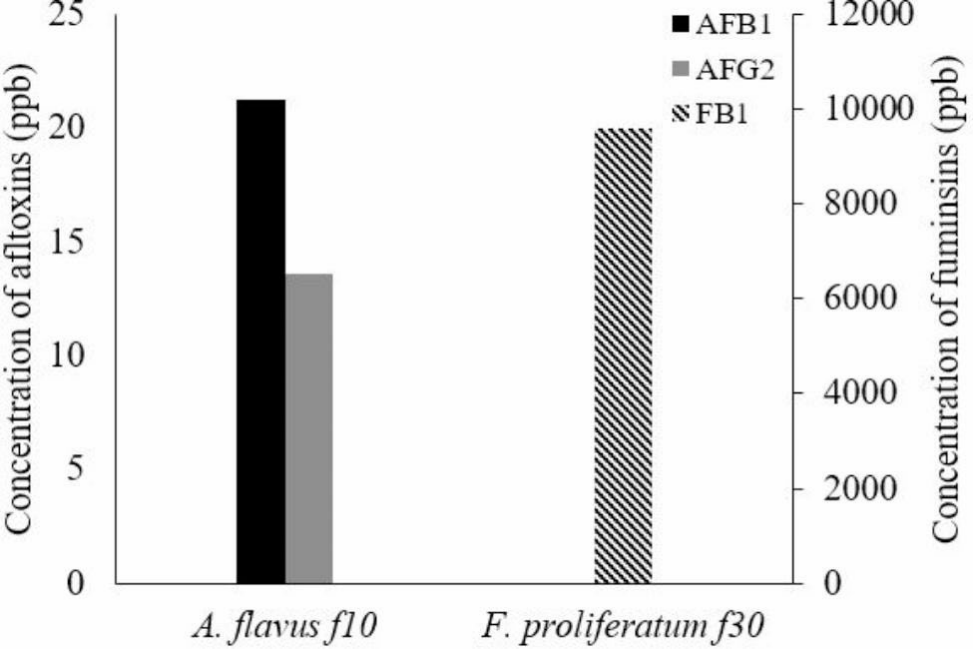
The concentration of the produced AFB_1_, AFG_2_ and FB_1_ toxins

High-performance liquid chromatography analysis was employed to figure out the concentrations of aflatoxins and fumonisin B_1_ produced by *A. flavus* f10 and *F. proliferatum* f30 strains, respectively. HPLC analyses continued for 15–25 min of retention time, but all the produced mycotoxins were recovered at the first 6 min of retention time (Fig. 1) and the tested fungal strains showed a high ability to produce mycotoxins. *A. flavus* f10 strain showed the ability to produce two types of aflatoxins namely, AFB_1_ and AFG_2_ in considerable concentrations of 21.239 ppb and 13.593 ppb, respectively. Additionally, *F. proliferatum* f30 strain showed a high degree of toxigenicity since it produces 9600 ppb of fumonisin B_1_ (Fig. 2).

### **The antagonistic effect of bacterial isolates against the toxigenic*A***. ***flavus*f10 and*F. proliferatum*f30 strains**

**Fig. 3:**
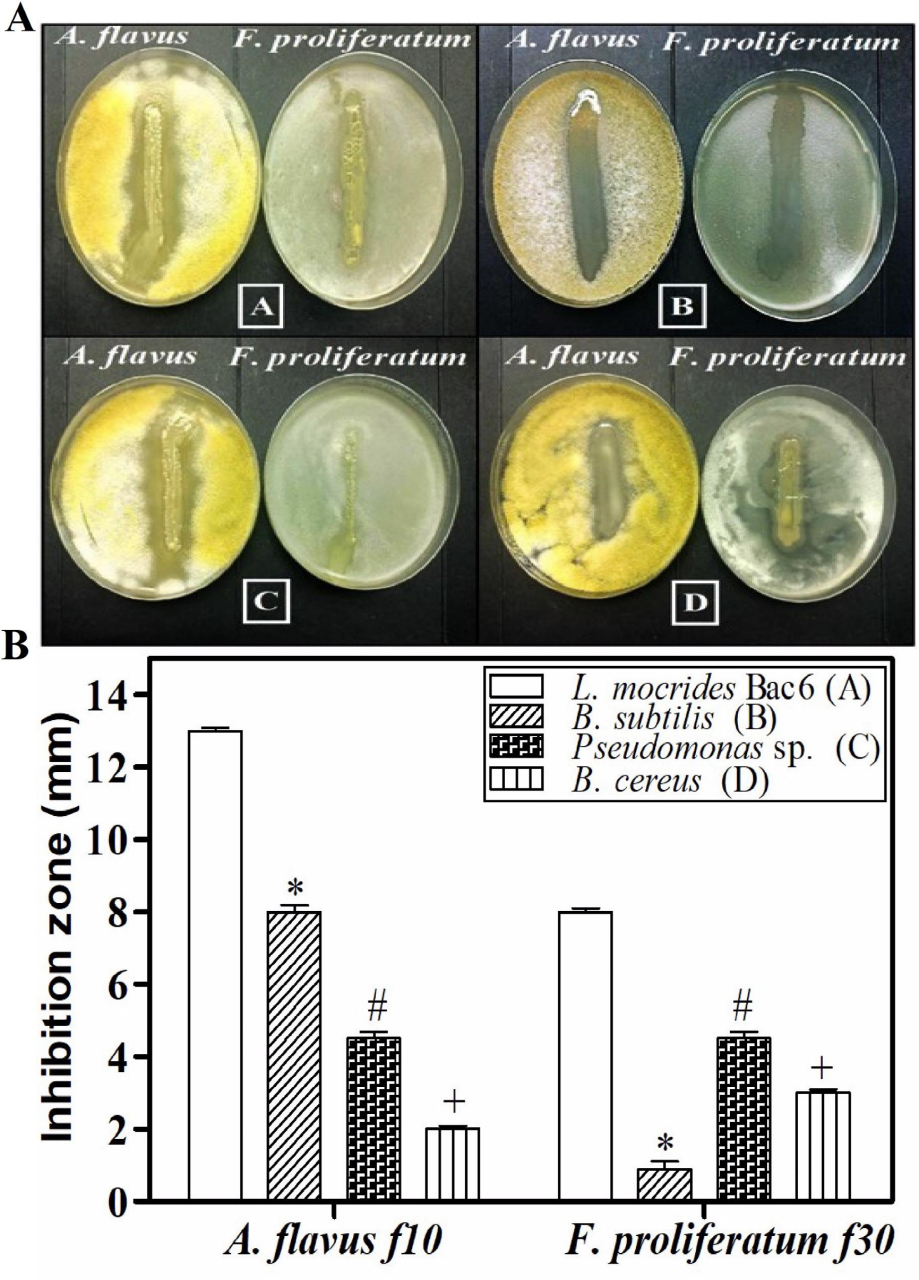
Antagonistic activities of bacterial isolates against toxigenic fungal isolates *A. flavus* f10, and *F. proliferatum* f30 recovered from corn and corn-based products. **(A)***Lysinibacillus macroides* Bac6, **(B)***Bacillus subtilis*, **(C)***Pseudomonas* sp. and **(D)***Bacillus cereus*. The sum of the data from three plates for each fungal strain are expressed as the mean ± SEM in *L. macroides* Bac6 (open bars), *Bacillus subtilis* (hatched bars), *Pseudomonas* sp. (herringbone bars) and *Bacillus cereus* (stripe bars). *P < 0.001, *L. mocrides* Bac6 vs. *B. subtilis*; ^#^P < 0.001, *L. mocrides* Bac6 vs. *Pseudomonas* sp.; and ; ^+^P < 0.001, *L. mocrides* Bac6 vs. *B. cereus*

Four bacterial isolates demonstrated various antagonistic activities against the two highest mycotoxin-producing fungal strains, *A. flavus* f10 and *F. proliferatum* f30. Bacterial isolate *Lysinibacillus macrolides* Bac6 exhibited the highest efficiency for inhibition of the growth of toxigenic fungi *A. flavus* f10 and *F. proliferatum* f30 recording a 13 mm and 8 mm inhibition zone, respectively. Whereas, bacterial isolates *Bacillus subtilis*, *Pseudomonas sp.* and *Bacillus cereus* showed inhibition zone of 8 mm, 4.5 mm and 2 mm, respectively against *(A) flavus* f10, as well as inhibition zone of 0.9 mm, 4.5 mm and 3 mm, respectively, against *F. proliferatum* f30 as shown in Table 1; Fig. 3A. Additionally, *Lysinibacillus macroides* Bac6 due to its highest efficacy among the four isolates as demonstrated in Fig. 3B. The accumulated data from three plates for each fungal strain are expressed as the mean ± SEM in *L. macroides* Bac6 (open bars), *Bacillus subtilis* (hatched bars), *Pseudomonas* sp. (herringbone bars) and *Bacillus cereus* (stripe bars). *P < 0.001, *L. mocrides* Bac6 vs. *(B) subtilis*; ^#^P < 0.001, *L. mocrides* Bac6 vs. *Pseudomonas* sp.; and ; ^+^P < 0.001, *L. mocrides* Bac6 vs. *B. cereus*.

**Table 1:** Antagonistic effect of bacterial isolates on toxigenic fungal isolates (A. *flavus* f10 and *F. proliferatum* f30) collected from corn and products containing corn. Bacterial isolate represents the four tested bacteria for the antagonist with the toxigenic fungi. Zone of inhibition presented in millimeter

Bacterial isolate	Inhibition zone (mm)	
	*A. flavus* f10	*F. proliferatum* f30
***Lysinibacillus_macrolides*** **Bac6**	13 ± 0.1	8 ± 0.1
***Bacillus subtilis***	8 ± 0.2	0.9 ± 0.2
***Pseudomonas sp.***	4.5 ± 0.2	4.5 ± 0.2
***Bacillus cereus***	2 ± 0.1	3 ± 0.1

### **Reduction of toxigenic fungal growth by*Lysinibacillus macroides*Bac6 strain**

**Fig. 4:**
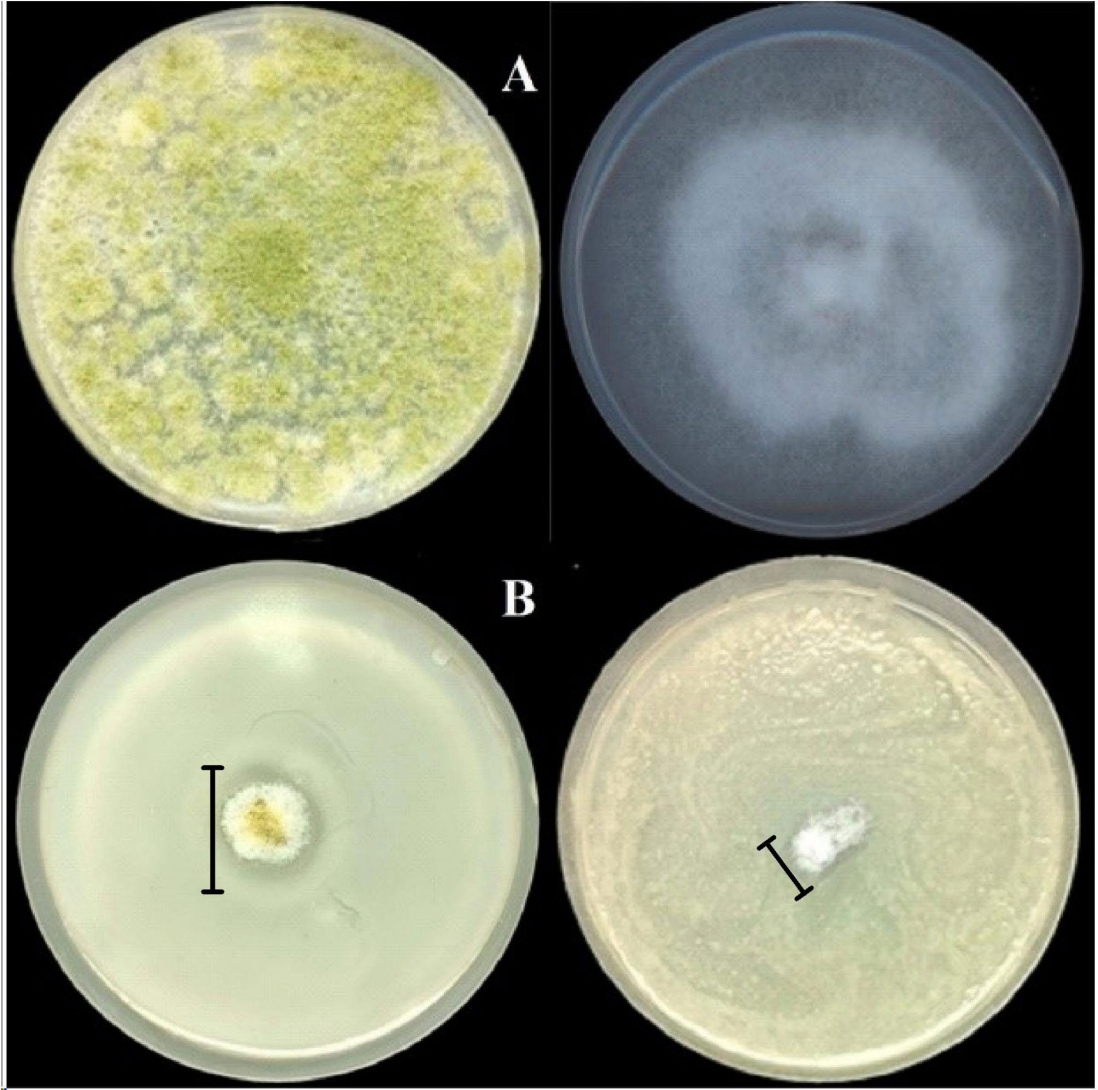
Spectrum of antifungal activities of *L. macroides* Bac6 against toxigenic *A. flavus* f10 and *F. proliferatum* f30 strains. The fungi in the first row **(A)** are the control (cultured fungi without bacterial effect), while those in the second row; **(B)** revealing substantial impacts on the development of the fungus in a dual culture after three days of incubation

The confirmation of the reduction of toxigenic fungal growth with the most active bacterial strain *Lysinibacillus macroides* Bac6 was assayed on a solid medium using a dual culture method. The highest antagonistic bacterial strains exhibiting inhibition percentage (%) of fungal growth compared to control fungi were 80 and 62.5% for *A. flavus* f10, and *F. proliferatum* f30, respectively (Fig. 4). Interestingly, the bacterial strain *L. macrolides* Bac6 is the most potent antagonistic bacteria against the tested mycotoxigenic-producing fungal strains *A. flavus* f10 and *F. proliferatum* f30. Therefore, it was selected for further studies to illustrate its crucial role in minimizing the toxins produced by toxigenic fungi.

Using morphological, microscopic, biochemical, and molecular methods, *L. macroides* Bac6 was identified. The partial 16 S rRNA gene sequence resulted in 931 bp; It was compared using a BLAST search (NCBI) with complete sequences that were available in the GenBank database. Sequences obtained with those retrieved from the GenBank database were subjected to Crustal analysis using Mega Align (DNA Star) for the phylogenetic analysis. Sequenced data were inserted in GenBank, and the resulting 16 S rRNA gene sequence showed identity similarity at 100% with *L. macroides* AzoM1 (MK942418.1), *L. macroides* JB_2 (MT197307.1) and *L. macroides* SMV311 (MN538917.1) (S2). Therefore, from the phylogenetic analyses, it can be identified as *Lysinibacillus macroides* Bac6 strain which, belongs to the family Bacillaceae, order Bacillales and class Firmicutes after which the resulting sequence was entered into the genebank with the corresponding accession number OQ080068.

### **Evaluation of the effect of*Lysinibacillus macroides*Bac6 strain on fungal growth of two toxigenic strains using electron microscopic examination**

**Fig. 5:**
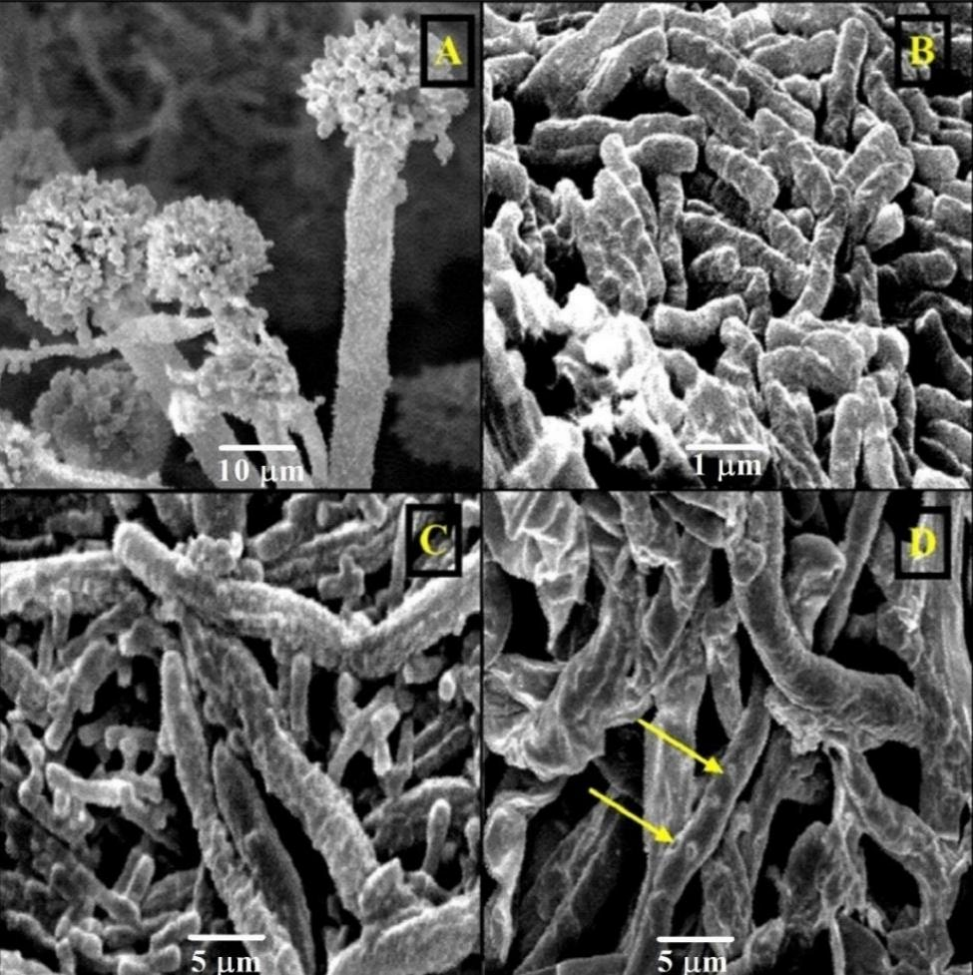
Scanning electron micrographs showed effect of *Lysinibacillus macroides* Bac6 on *Aspergillus flavus* f10 fungal growth. **(A)***Aspergillus flavus* f10 growth “control”, **(B)***Lysinibacillus macroides* Bac6 cells. **(C**, **D)** bacterial colonization around fungal hyphae, deformations, pitting and inhibition of sporulation

**Fig. 6:**
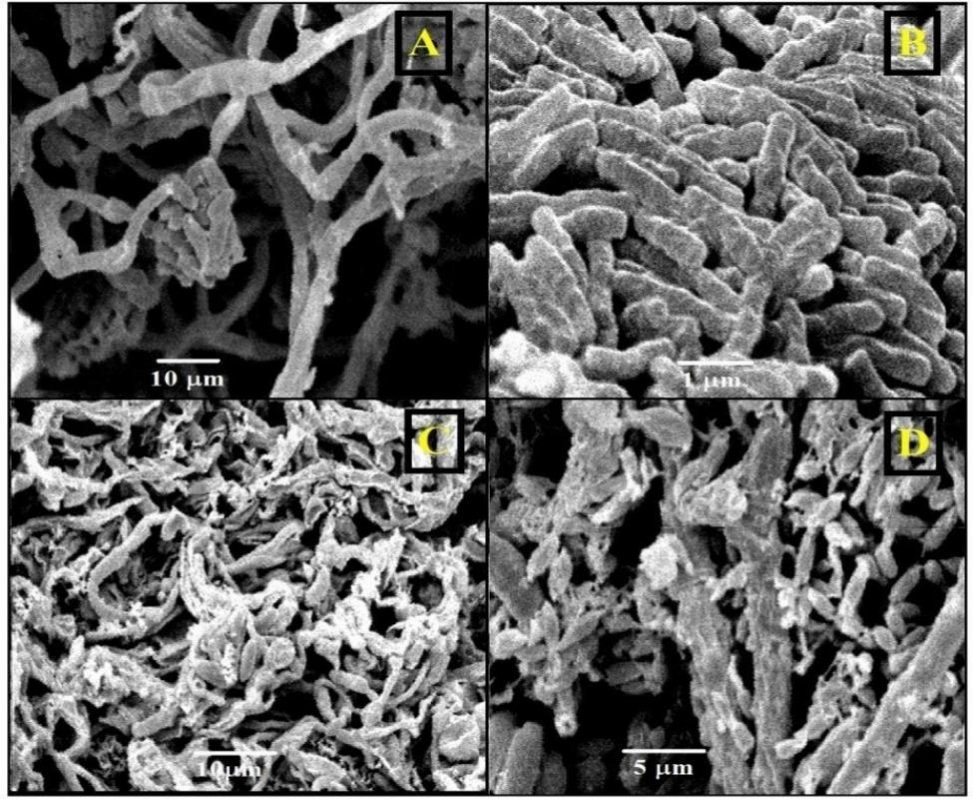
Scanning electron micrographs showed effect of *Lysinibacillus macroides* Bac6 on *Fusarium proliferatum* f30 fungal growth. **(A)***Fusarium proliferatum* f30 growth “control”, **(B)***Lysinibacillus macroides* Bac6 cells. **(C**, **D)** bacterial colonization around fungal hyphae, deformations, pitting and inhibition of sporulation

The impact of the highest antagonistic bacterial strain (*Lysinibacillus macroides* Bac6) on the growth of toxigenic fungi was evaluated using scanning electron microscope (SEM) to evaluate the possible mode of action of *Lysinibacillus macroides* Bac6 (Figs. 5B and 6B) against *Aspergillus flavus* f10 (Fig. 5A) and *Fusarium proliferatum* f30 (Fig. 6A). The resulting SEM graphs showed significant bacterial colonisation and persistent adhesion around the hyphae of toxigenic mould (Fig. 5C & 6C). The colonized hyphae displayed severely malformed and limited proliferation (Figs. 5D and 6D), pitting and damaged appearance of the hyphal cell wall compared with control fungal growth. As well as, it was noticed that, there is strong inhibition of conidia formation.

### Chitinase activity

**Fig. 7:**
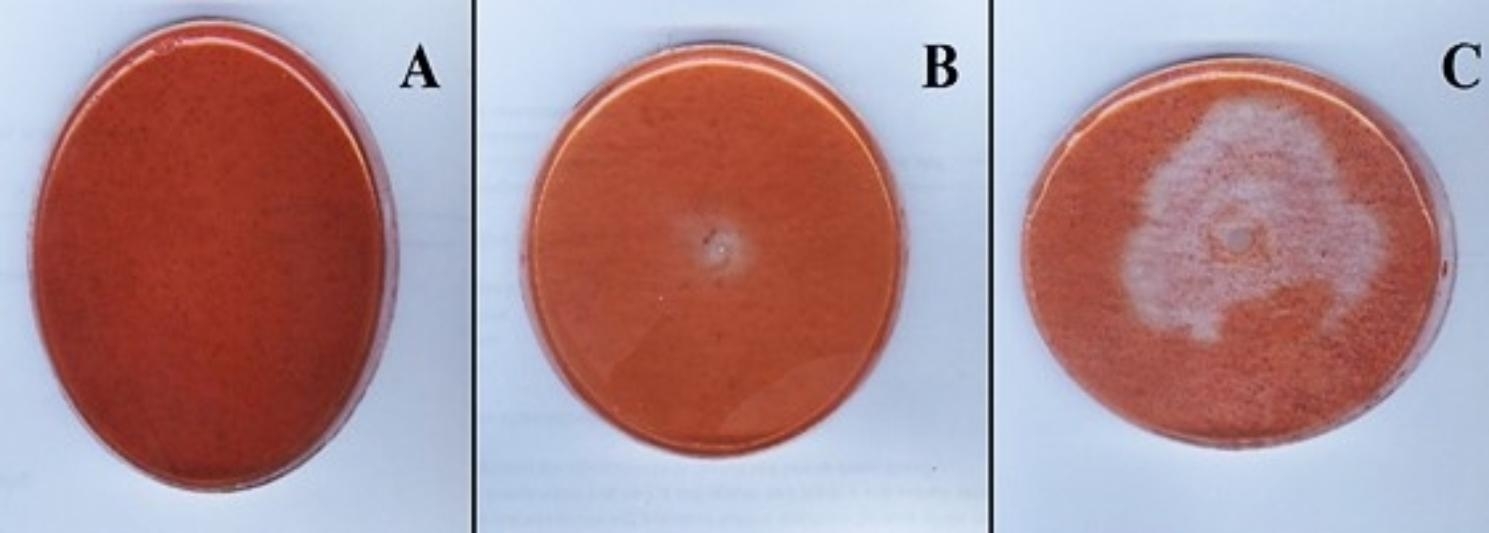
Chitinase activity of *Lysinibacillus macroides* on solid medium. **(A)** control plate floded with Congo red, **(B)** chitinase activity of bacterial strain (clear zone) after incubation for 3 days and **(C)** chitinase activity of bacterial strain (clear zone) after incubation for 5 days

Scanning electron microscope (SEM) graphing showed pores formation of the toxigenic fungal hyphae when grown with *L. macroides* Bac6 strain in dual culture. Therefore, it was expected that the bacterial cells had the capability to produce hydrolyzing enzymes that induced fungal cell wall hydrolysis and pore formation by chitinase. The bacterial strain (*L. macroides* Bac6) was tested for chitinase enzyme production. Control without bacterial colony (Fig. 7A). It showed the capability to produce chitinase, recording the appearance of a clear zone of 12 mm after 24 h (Fig. 7B). Interestingly, it showed 55 mm inhibition zone on a solid chitin medium after 3 days incubation period (Fig. 7C). Moreover, using spectrophotometer, the highest antagonistic bacterial strain showed chitinase productivity of 26.45 U/L and chitinase activity of 0.12 U/mL/min. after 5 days incubation period.

### **Composition of the metabolites profiling of*L. macroides*Bac6**

**Fig. 8:**
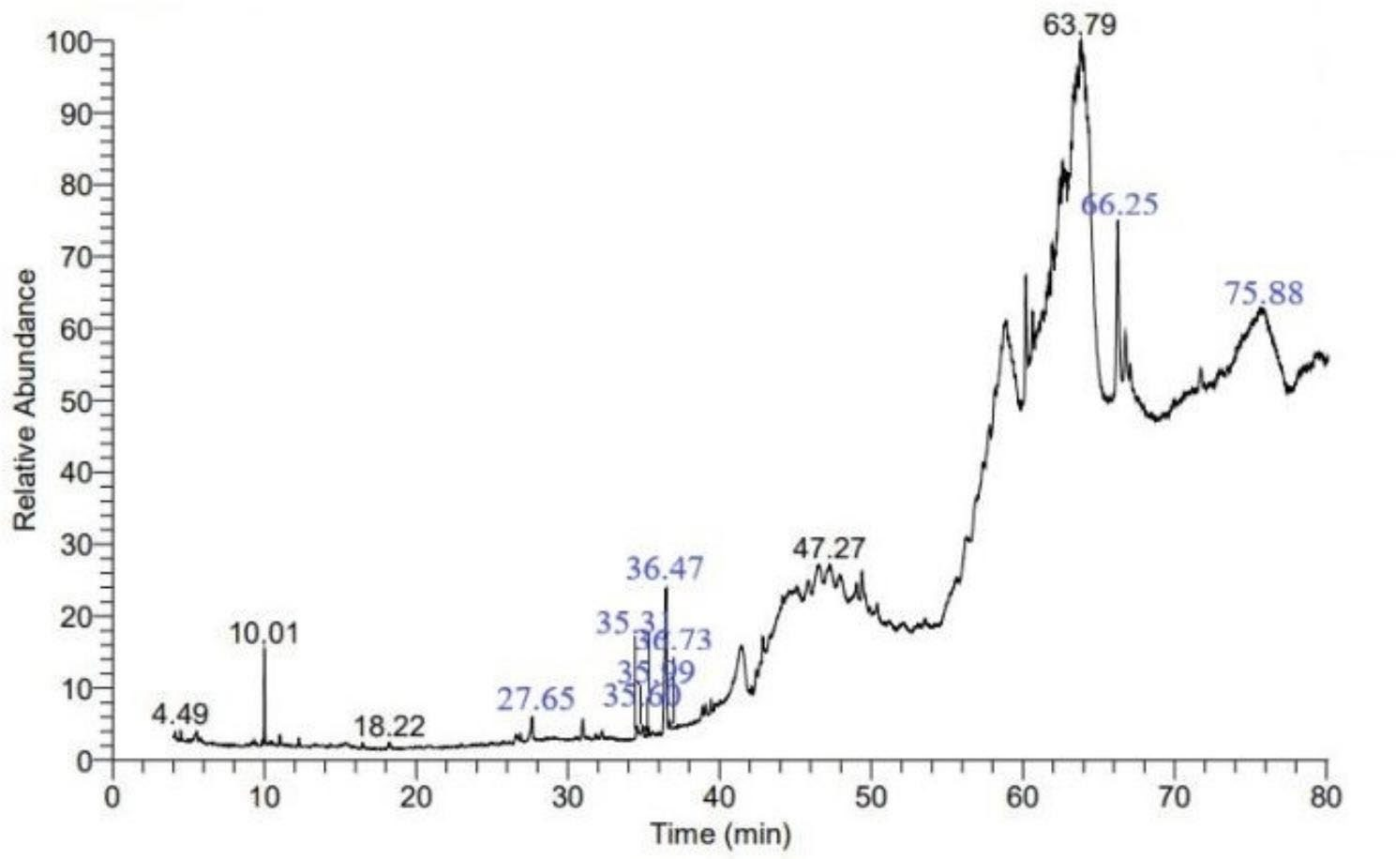
GC − MS chromatograph of the detected compounds in *L. macroides* Bac6 strain extract

The bacterial metabolites analyses were performed using Gas Chromatography/Mass Spectrometry (GC-MS), to assay the bioactive compounds produced by the antagonistic bacterial strain that may be responsible for the inhibition of the growth of toxigenic fungi. The GC-MS analysis revealed that the most common bacterial metabolites were Pyrrolo[1,2-a] pyrazine-1,4-dione, hexahydro (6.69%), 9,12,15-Octadecatrienoic acid,2,3-bis[(trimethylsilyl)oxy]propyl ester, (Z,Z,Z)- (6.57%), Hexadecenoic acid, 1-(hydroxymethyl)-1,2-ethanediyl ester (1.65%), Glycerol 2-acetate 1,3-dipalmitate (1.14), Ethyl iso-allocholate (1.07%). Additionally, 2(3 H)-furanone,5-heptyldihydro- (0.81%) of the total analytes. Whereas, the bacterial metabolites Agaricic acid (0.42%), Digotoxin (0.42%), N, N’-Bis (Carbobenzyloxy)-lysine methyl(ester) (0.33%), Oleic acid (0.29%), 2-Myristynoyl pantetheine (0.26%), 2(3 H)-Furanone, 5-heptyldihydro- (0.18%) and hexadecenoic acid methyl ester (0.15%) were detected in the GC-MS analysis (Fig. 8; Table 2).

**Table 2:** Major compounds of *L. macroides* Bac6 strain metabolites detected by Gas Chromatography-Mass Spectrometry

No.	COMPOUND	RT.	M.wt	% of total analytes
1	Pyrrolo[1,2-a] pyrazine-1,4-dione, hexahydro	36.47	154	6.69
2	9,12,15-octadecatrienoic acid,2,3 bis[trimethylsily] propylester, (Z, Z, Z)-	66.25	496	6.57
3	Benzyl chloride	10.01	126	2.33
4	Hexadecenoic acid, 1-(hydroxymethyl)-1,2-ethanediyl ester	47.27	568	1.65
5	Glycerol 2-acetate 1,3-dipalmitate	63.79	610	1.14
6	Ethyl iso-allocholate	75.88	436	1.07
7	2(3 H)-furanone,5-heptyldihydro-	27.65	184	0.81
8	Agaricic acid	35.60	416	0.42
9	Digotoxin	35.31	764	0.42
10	N, N’-Bis (Carbobenzyloxy)-lysine methyl(ester)	4.49	428	0.33
11	Oleic acid	36.73	282	0.29
12	2-Myristynoyl pantetheine	18.22	484	0.26
13	2(3 H)-Furanone, 5-heptyldihydro-	27.65	184	0.18
14	hexadecenoic acid methyl ester	35.99	270	0.15

The data in the table explains the range of various fourteen bioactive chemical components at various concentrations 0.1 of the total metabolites of the L. macroides Bac6 strain that were recovered throughout various retention times. Retention time every minute (RT); Compound: Active substances identified by GC-MS; (%): Compound percentage; M. wt.: Compound molecular weight

### **Impact of*L. macroides*Bac6 strain on the formation of mycotoxins**

**Fig. 9:**
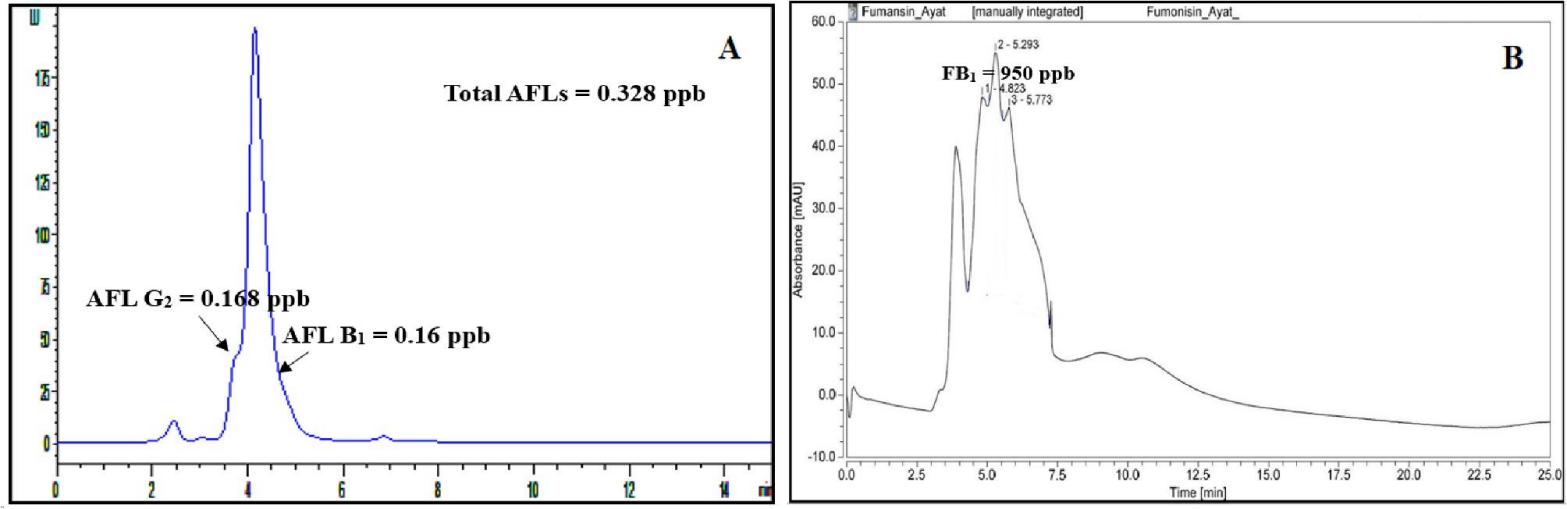
Chromatograms of aflatoxins production by *Aspergillus flavus* f10 **(A)** and Fumonisin B_1_ production by *Fusarium proliferatum* f30 **(B)** after exposure to bacterial strain (*L. macroides* Bac6)

The data from HPLC for each fungal extract with their toxins and the fungal extracts with their toxins treated by L. macroides are expressed as the concentration (ppb) in Table 3. *P < 0.0001, A. flavus f10 (AFB1) + L. macroides vs. A. flavus f10 (AFB1); # P < 0.0001, A. flavus f10 (AFG2) + L. macroides vs. A. flavus f10 (AFG2); and ; +P < 0.0001, F. proliferatum f30 (FB1) + L. macroides vs. F. proliferatum f30 (FB1). The resulted data revealed that the active bacterial cells had a significant reduction for aflatoxin B_1_ and aflatoxin G_2_ which were produced by *Aspergillus flavus* f10 recording 99.25% and 99% inhibition, respectively. In addition, the *Lysinibacillus macroides* showed strong inhibition of fumonisin B_1_ (90% inhibition) produced by *Fusarium proliferatum* f30 (Fig. 9).

**Table 3:** Effect of *Lysinibacillus macroides* on mycotoxins produced by toxigenic fungal isolates

Fungus ± Treatment	Mycotoxin	Concentration (ppb)
*A. flavus* f10	AFB_1_ AFG_2_	21.239 11.666
* A. flavus* f10 *+ L. macroides*	AFB_1_ AFG_2_	0.16^*^ 0.168^#^
*F. proliferatum* f30	FB_1_	9600.91
* F. proliferatum* f30 *+ L. macroides*	FB_1_	950^+^

The data from HPLC for each fungal extracts with their toxins and the fungal extracts with their toxins treated by L. macroides are expressed as the concentration (ppb). *P < 0.0001, A. flavus f10 (AFB_1_) + L. macroides vs. A. flavus f10 (AFB_1_); # P < 0.0001, A. flavus f10 (AFG_2_) + L. macroides vs. A. flavus f10 (AFG_2_); and ; +P < 0.0001, F. proliferatum f30 (FB_1_) + L. macroides vs. F. proliferatum f30 (FB_1_)

## Discussion

Multiple lines of evidence in vitro were employed to show that fumonisin and aflatoxins accumulations reflect the mycotoxigenic risk when the maize is infected with toxic *Fusarium* section *Liseola* and *Aspergillus* section *Flavi* [47]. Similarly, Izzati et al. [48] shown that *F. proliferatum* was found on nearly all maize farms. Other lines of evidence on corn were investigated and demonstrated the high occurrence of *A. flavus* [49, 50], as well as *Fusarium* species especially *Fusarium graminearum* and *Fusarium proliferatum* were very common in all maize-cultivated areas [51]. *A. flavus*, is the most risky worldwide species that is easily able to colonize corn [52]. Contamination with mycotoxins and their producers in maize and products based on corn is mainly caused by aflatoxins, fumonisins, and their primary fungal producers [53]. Regarding this *F. proliferatum*, *F. verticillioides* produced FB1 and FB2, with FB1 serving as the primary analogue (representing 75% of the total fumonisins). These isolates are thought to yield more than 500 g/g of FB1 [54]. Our results revealed that the potent toxigenic fungal strains *A. flavus* f10 and *F. proliferatum* f30 which are isolated from corn samples refered that strict quarantined and proper storage practices recommended to be used with imported goods in order to minimize infection with toxigenic moulds and steer clear of hazards to animal and human health.

The results were obtained from the HPLC method was employed to verify and quantify the aflatoxins (AFB_1_ – AFG_2_), and fumonisin B_1_ generated by the highest concentrations of active aflatoxins and strains that produce fumonisins, respectively, namely *A. flavus* f10, and *F. proliferatum* f30. Our data revealed that, *Aspergillus flavus* f10 that was invading cornflakes could produce aflatoxin B_1_ 21.239 ppb and aflatoxin G_2_ 13.493 ppb and *F. proliferatum* f30 produce 9600 ppb of fumonisin B_1_. Compared to another previous study that stated that *A. flavus* isolates could produce aflatoxin B_1_ in the range of 0.09–50.68 ppb and aflatoxin B_2_ 0.33–9.2 ppb isolated from Malaysian Sweet Corn [55]. In other research, levels of Aflatoxins secreted by *A. parasiticus* and *A. flavus* isolated from silage of corn ranged 2–45 ppb and 2–100 ppb, respectively [56]. In addition, a study performed on corn and popcorn samples monitored that *A. flavus* var. *columnaris* and *A. flavus* isolates could produce aflatoxins with range of 1–8 ppb [57]. *F. proliferatum* and *Fusarium verticillioides* were investigated to accumulate FB_1_ and FB_2_ in maize kernels, which could result in a harmful or economical crop loss [58]. It is critical to find strategies to safeguard the maize crop from fumonisin building up. In the study performed [58], the production of fumonisin B_1_ is 6.22 ± 0.89 ppb. Similarly, Mohamed et al., published that FB_1_ was found in all silos samples and corn markets with a range of 13.69-175.54 ppb [59].

Our current study focused on the potentiality of using a biological control agent to reduce the growth of *A. flavus* f10 and *F. proliferatum* and their toxins. Our results revealed that *L. macrolides* Bac6 was the best one among all the tested bacterial isolates, due to its highest efficacy as demonstrated in Fig. 3B, which interpreted the data presented in Table 1. Although, there is no significance among the effects of the four tested bacterial isolates against both tested mycotoxigenic fungi. We demonstrated that the most antagonistic is *L. macrolides* Bac6, as a result of its safety as demonstrated in several research papers using it as a biological agent against pathogens [41, 60, 61]. Therefore, the *Lysinibacillus macrolides* Bac6 was the best choice to be used as a biological control agent. The proportion of fungal growth inhibited by *Lysinibacillus macroides* Bac6 as compared to control fungi was 80 and 62.5% for *A. flavus* f10 and *F. proliferatum* f30, respectively. As mentioned before in a previous study *Bacillus megaterium* BM344-1 has the ability to reduce the toxigenic fungi growth. The inhibition ratios (%) of *P. verrucosum, (A) flavus*, and *F. verticillioides* outperformed control fungi by 66.7, 29.4, and 18.2%, respectively [62]. Zeidan et al. [63] The most sensitive to yeast VOCs was found to be *Penicillium* followed by *Aspergillus*, whereas *Fusarium* was found to be the least sensitive. A key factor in fungus resistance is the nature of the fungal cell wall, which is affected by stressors in the microenvironment. The antagonistic effects of *Bacillus* volatiles as *(B) subtilis*, *Bacillus amyloliquefaciens*, *Bacillus cereus*, and *B. megaterium* towards toxigenic and phytopathogenic *Penicillium* and *Aspergillus* spp. have been illustrated [62, 64, 65].

We evaluated the underlying mechanism of the antagonistic effect of *L. macrolides* via electron microscopic examination, and we detected that the effect may be attributed to the production of chitinase enzyme. The tested *Lysinibacillus macroides* Bac6 strain in this study showed high potential production of exo-chitinase enzyme. According to the study that illustrated that *Lysinibacillus* spp. also produces some of these types of antimicrobial compounds, which include the development of cell wall-degrading enzymes as a biocontrol approach [40]. Cell wall-degrading enzyme-producer *Lysinibacillus* can inhibit fungal hyphal development [40].

Bacterial metabolites analysis performed by gas chromatography − mass spectrometry (GC − MS) revealed the presence of several compounds including Pyrrolo[1,2-a] pyrazine-1,4-dione, hexahydro. This compound was the highest prevalent in the *L. macroides* Bac6 strain metabolites. Interestingly, it was reported as a bactericidal compound which also, isolated from *Bacillus tequilensis* MSI45 against multidrug-resistant pathogenic *Staphylococcus aureus* [66]. Moreover, it has been shown that *Microcystis aeruginosa* is susceptible to algicides, which is a compound was detected in *Bacillus* Lzh-5 [67]. Furthermore, GC-MS analysis of *L. macroides* Bac6 strain metabolites revealed that the presence of these bioactive compounds namely, Pyrrolo[1,2-a] pyrazine-1,4-dione, hexahydro 2(3 H)-furanone,5-heptyldihydro, 9,12,15-octadecatrienoicacid,2,3 bis[trimethylsily] propylester, (Z, Z, Z)-, agaricic acid, digotoxin, ethyl iso-allocholate, oleic acid and hexadecanoic acid methyl ester. All compounds are well-known microbial biomolecules that have strong antagonistic activities against toxigenic as well as phytopathogenic fungi [68] [69] [70] [71]; [62, 72] [66]. While 9,12,15-octadecatrienoic acid, 2,3-bis[(trimethylsilyl)oxy]propyl ester, (Z,Z,Z)- (6.57%) was the second molecule found with a large peak area and has been linked to antimicrobial activity [73], hexadecenoic acid has recently been linked to antifungal activity [74]. In addition, the 1-(hydroxymethyl)-1,2-ethanediyl ester (1.65%) had antifungal action [75], as did Glycerol 2-acetate 1,3-dipalmitate (1.14) [76], and Ethyl iso-allocholate (1.07%). Furthermore, 2(3 H)-furanone,5-heptyldihydro- (0.81% of total analytes) demonstrated antifungal activity. [77–79].

Aflatoxin and fumonisin-producing fungi share the same habitat as other microorganisms that can influence toxin production [80, 81]. Our results revealed that *Lysinibacillus macroides* Bac6 produced an inhibitory effect on *A. flavus* f10 and *F. proliferatum* f30 growth and their toxin production. Surprisingly, the *Lysinibacillus macroides* Bac6 metabolites inhibited AFB_1_ and AFG_2_ which produced by *Aspergillus flavus f10*, and FB_1_ that produced by *Fusarium proliferatum* f30, with 99.25, 99, and 90%, respectively. Saleh et al. [62] reported that aflatoxins (AFB1, AFG1, and AFG2), ochratoxin A, and FB1 production on artificial medium were completely inhibited after exposure to (A) flavus, P. verrucosum, and F. verticillioides to Bacillus megaterium BM344-1 VOCs. Moreover, Pereira et al. [81] study demonstrated that *(B) amyloliquefaciens* were significantly reducing FB_1_ and FB_2_ levels. Furthermore, it has been documented that volatiles released by *B. megaterium* KU143 and *B. licheniformis* 350-2 on un-hulled rice and maize ears prevent the production of aflatoxins by *A. flavus* [82]^,^ [83], respectively.

## Conclusion

**Fig. 10:**
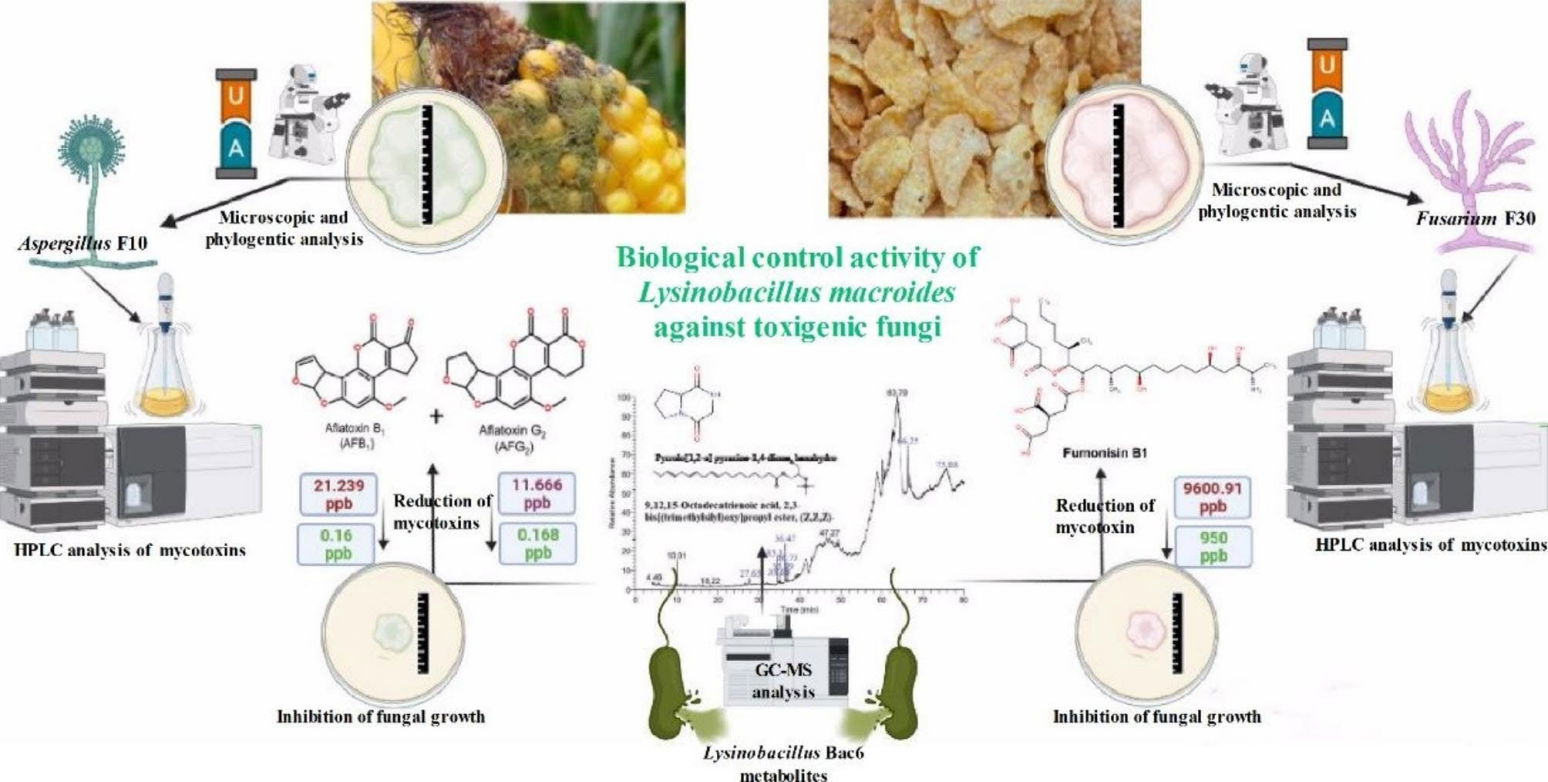
To summarize our study, we started with the isolation of the toxigenic fungi from corn samples and corn-based products, separation of the toxin after identification of the toxigenic fungi to be analyzed via HPLC to detect the types of mycotoxins, and finally illustrating the biological control effect of bacterial metabolites detected with GC-MS against those toxigenic fungi.

In conclusion, mycotoxins pose a serious threat to the safety of food, especially in low- and middle-income nations, incurring financial and human health consequences. Bacterial strains can be used as an eco-friendly, non-chemical, and low-cost biological control method. For the first time, we investigated the efficacy of using *L. macroides* Bac6 as a biological control agent against the most prevalent mycotoxigenic fungi invading corn and corn-based products through the potential impact of bacterial metabolites that significantly reduced AFB_1_ and AFG_2_ produced by *A. flavus* f10 and strongly inhibited fumonisin B_1_ produced by *F. proliferatum* f30. To better summarize, the methods and the resulted data of our study were demonstrated in (Fig. 10).

## Materials and methods

### Toxigenic fungi

*Aspergillus flavus* f10 and *Fusarium proliferatum* f30 strains were obtained from our previous work, in which they were isolated from yellow-corn and cornflakes, respectively [46]. According to our published paper, prior to usage, the tested fungal isolates were cultivated and cultured on potato dextrose agar (PDA) at 28 °C. [45, 84]. Both strains were identified by molecular characteristics based on 18 S rRNA.

### Analyses of mycotoxins production by HPLC technique

The fungal inoculum preparation was carried out using freshly grown fungal cultures, seven days old, at 28 °C. A 1 cm disc was removed from the borders of the fungal growth using a cork-porer and utilized as fungal inoculum according to [85]. As an enhanced medium for mycotoxins formation, liquid yeast extract sucrose medium (YES) was employed containing (g/L); yeast extract, 1; peptone, 10; K_2_HPO_4_,1; MgSO_4_.7H_2_O, 0.5; sucrose, 30; KCl, 0.5; FeSO_4_.H_2_O, 0.01; and NaNO_3_, 2.0, and the pH was adjusted to 6.5. In 100 ml capacity conical flasks, 25 ml of the medium (YES) was pipetted, autoclaved, inoculated then incubated at 28 °C for two weeks and three weeks under static conditions for aflatoxins and fumonisin B_1_ production, respectively. For aflatoxins extraction, the homogenization occurred in the fungal broth. Using a fast speeds blender, mix 25 millilitres of chloroform solvent for a period of five minutes. The aqueous phase was subsequently filtered using Whatman filter paper No.1 after the organic phase was separated from it using a separating funnel, then dehydrated over a solution of anhydrous sodium sulphate, and dried to near dryness on a rotating evaporator. Each extract’s residuals were then reconstituted in two millilitres of chloroform then preserved in tiny brown bottles until the detection process. In contrast, the acetonitrile technique (5 ml/g of culture media) reported by [86] has been selected for fumonisin B_1_.

The mycotoxins were subjected to a quantitative estimation using high-performance liquid chromatography (HPLC) according to [87]. In the case of aflatoxins determination, the mobile phase comprised a combination of water, acetonitrile, and methanol (55:30:15 v/v/v), whereas, for fumonisin B_1,_ two mobile phases have been used, namely solvent A (water: acetonitrile: acetic acid (59:4:1 v/v/v)) and solvent B acetonitrile: acetic acid (99:1 v/v). Aflatoxins and fumonisin B_1_ were detected using a detector that detects fluorescence with wavelengths of excitation of 295 and 335 nm, respectively, and emitting wavelengths of 330 and 440 nanometers, respectively [88–90]. The studies were carried out using HPLC system (Agilent Technologies Series 1200, G1321A FLD with column Zorbax, the Eclipse programme + C18) located at Assuit University’s Analytical Chemistry Unit.

### Investigating the antagonistic activities of bacterial isolates against the toxigenic fungi

The antagonistic activities of the recovered bacterial isolates on the highest toxin-producing fungal strains (*A. flavus* F10 and *F. proliferatum* F30) were tested in vitro. The bacterial isolates recovered from soil were streaked with a line method at the center on PDA plates with a toxigenic strain of fungus inoculum, along with a 7-day incubation period at 28 °C for the cultures. Each treatment included three replications. The inhibitory zone was identified at the end of of the incubation time [91, 92].

*Lysinibacillus macroides* Bac6, the highest antagonistic bacteria, were selected for assay of the reduction of toxigenic fungal growth. Well-grown bacterial colonies which were incubated at 35 º C for 48 h were picked and further purified by streaking [93, 94]. The isolates were maintained on nutrient agar (NA) slants and stored at 4 ºC. The bacterial cultures were identified based on morphology (shape, Gram stain, spore formation and motility). *L. macroides* Bac6 has been identified by molecular characteristics based on 16 S rRNA [95].

### **Determination of the reduced level in toxic fungal growth by antagonistic bacteria*Lysinibacillus macroides*** Bac6 strain

The decrease in the development of toxic fungi (*A. flavus* f10 and *F. proliferatum* f30) by *Lysinibacillus macroides* Bac6 strain was conducted according to the method described in [89, 92]. The interaction between the tested bacterial strains and toxigenic strains was assessed using PDA. 1 mL of bacterial suspension (10^7^ CFU/mL) was mixed with 15 mL of molten PDA medium before solidification and poured into Petri dishes (90 mm diameter). After solidification of the medium, mycelial discs with 10 mm diameter were cut from the fungal active growing margins of 7 days old cultures and placed at the center of the agar surface. The control plates were inoculated with tested fungi but without bacteria following the same procedure. All plates were incubated at 28 °C for 72 h. The experiment was carried out in triplicates. Fungal growth inhibition was calculated by measuring the diameter of the fungal colonies using the following equation:

$$\text{F}\text{u}\text{n}\text{g}\text{a}\text{l} \text{G}\text{r}\text{o}\text{w}\text{t}\text{h} \text{I}\text{n}\text{h}\text{i}\text{b}\text{i}\text{t}\text{i}\text{o}\text{n} \left(\text{\%}\right)=\frac{\left(\text{C}-\text{T}\right)}{C}100$$

Where C is the diameter of the fungal colonies in control plates and.

T is the diameter of the fungal colonies in treated plates.

The alterations of fungal growth which was caused by antagonistic bacterial strains were shown and confirmed by scanning electron microscopy (SEM).

### Evaluation of the impact of antagonistic bacterial strain on the Toxigenic fungal growth was evaluated by using scanning electron microscopy (SEM)

To evaluate the antagonistic capability of *L. macroides* Bac6 strain against the toxigenic fungi, the fungal mycelia of dual culture were examined using SEM. In brief, A 10-mm disc of mould growth boundary was removed and fixed for 2 days in 5% cool buffer glutaraldehyde. The samples were then rinsed three times (30 min each) with sodium cacodylate buffer before being post-fixed in 1% osmium tetroxide for two hours. The samples were then rinsed three times in the same buffer (30 min each) and dehydrated using an escalating ethanol gradient (30%, 50%, 70%, and 90%) for two hours and 100% ethanol for two days, before being treated with amyl acetate for a further two days. Following that, the samples were dried in a critical point drainer with liquid carbon dioxide before being attached to a metallic block with silver paint [91, 96].

### **Assay of chitinase activity of*L. macroides*Bac6 strain**

#### Preparation of colloidal chitin

According to Hussin and Ab Majid, a colloidal of chitin was manufactured. Briefly, five grams powder of chitin were combined in a beaker with conc. Hydrochloric acid (~ 10 M HCl) 60 ml. Via a rod of glass, the prepared mixture was continuously stirred for a period of 5 min then 1 min gently stirring at a time interval 5 min for 1 h. In a 2 L conical flask, the chitin-HCl combination was subsequently processed using 2 L of cold distilled water and then it was kept under static conditions for 12 h at 4 ºC. The precipitation was gathered using crossing two distinct phases of until the colloidal chitin reached a pH of 7, the filter cloth was continuously rinsed with normal water. The produced colloid of chitin was squeezed between the filter paper (to reduce any residual moisture) and subsequently kept at 4 °C until it was used again [97].

#### Chitinase production of *L. macroides* Bac6 strain on solid medium

The initial screening was carried out by adding 10 L of a 24-hour-old culture of *L. macroides* Bac6 (10^7^ CFU/mL) to the middle of NA plates that contained 1% colloidal chitin, then incubating those plates at 30 °C for 5 days. Congo red (1%) was used to demonstrate the activity of the enzyme for 30 s, after which the dye was fully removed with a solution of sodium chloride (30 g L^-1^) until a zone of transparency established as a result of chitin hydrolysis. The test was performed in three replicates. [98].

#### **Chitinase enzyme activity of*L. macroides*Bac6 strain on liquid medium**

A 250 mL Elementary flask with 100 mL NA and 1% colloidal chitin was filled with 10 mL of a 24-hour-old cultures of bacteria. The incubation was conducted on a rotating shaking device for five days at 30 °C and 180 rpm. The supernatant without cells was obtained by centrifuging the culture broths at a speed of 120 rpm for 10 min at 4 °C.

Applying the dinitrosalicylic acid (DNS) method, the efficacy of chitinase was assessed by looking for sugars that reduced after the enzymatic process. The test was performed with the specified methodology. [97]. After being produced in a 50 mM phosphate buffer (pH 7.0) with just one millilitre of crude extract of enzyme (cell-free culture supernatant), the substrate mixture consisted of 1% colloidal chitin. The resulting mixture had been warmed in a water bath at 37 °C for a period of one hour. By adding 3 mL of DNS reagent and boiling it in water for 20 min, the process was halted. The mixture was centrifuged at 5000 rpm for five minutes after cooling. The wavelength of the absorbance has been identified at 540 nm using the Model T60 UV-VIS spectrophotometer. The amount of chitinase activity (U/mL) was calculated as the amount of enzyme that, under normal test conditions, generated 1 mol of dinitrosalicylic acid per minute [99]. The experiment was carried out in triplicate.

### **Assessment of*L. macroides*Bac6 metabolites using GC-MS analyses**

Tryptic soy broth (TSB) was used to culture the bacterial strain. The following ingredients were present in tryptic soy broth in grammes per litre: 17.0 for pancreatic digest of casein; 3.0 for papaic digest of soybean meal; sodium chloride, 5.0; dextrose, 2.5; and dibasic potassium phosphate, 2.5; pH after sterilisation was 7.30.2. Following a 24-hour incubation period at 30 °C, the bacterial culture was centrifuged for 5 min at 6000 g before being filtered through a sterile 0.22 micron membrane filter. With petroleum ether (1:1 v/v), the bacterial metabolites were separated from the resultant supernatant.

To quantify the bacterial metabolites, the GC/MS (Model: DPC-Direct Probe Controller (DPC20451), Thermo Scientific,USA; at the Chemistry Department, college of Science at Assiut University was used. A capillary column TG-5MS with dimensions of 30 m, 0.25 mm i.d., and 1 m film thicknesses was used for the separation of fatty acid ester compounds. The temperature of the oven was initially maintained at 80 °C for 5 min, and then increased at a ramp rate of 10 °C/min to 150 °C for 10 min, 200 °C at a ramping speed of 10 °C/min (held for 10 min), and 250 °C at a ramp rate of 5 °C/min (held for 13 min). Helium was employed as a carrying gas at a flow rate of 0.5 mL/min, and the split flow was 10 mL/min. [100, 101].

### **Effect of*Lysinibacillus macroides*Bac6 on mycotoxins production**

Minimization of toxins production by toxigenic fungi by using active bacterial cells was tested by growing the fungus for 10 days in coculture with the bacterial living cells (10^7^ CFU/ml), from 24 h old culture [90, 98]. As previously mentioned, high-performance liquid chromatography (HPLC) was used to extract the poisons and quantify their amounts.

### Statistical analysis

GraphPad Prism software version 5 was used to conduct statistical analysis on normally distributed data, which are represented as means standard errors of the means (SEM). One-way ANOVA was used to analyse the significant differences between the three groups, and Tukey’s posttest followed.

### Electronic supplementary material

Below is the link to the electronic supplementary material.

Supplementary Material 1

## Data Availability

All the generated data and the accession number for all the tested microorganisms are available and included in the manuscript.
