# Supplementary material for: Antifungal activity of Lysinibacillus macroides against toxigenic Aspergillus flavus and Fusarium proliferatum and analysis of its mycotoxin minimization potential
Source: BMC Microbiol. 2023 Sep 26;23:269. doi: 10.1186/s12866-023-03007-4 (PMC10521556; doi:10.1186/s12866-023-03007-4)
Supplement: Supplementary file 1 — Supplementary Material 1 [file 12866_2023_3007_MOESM1_ESM.docx]

**Antifungal Activity of *Lysinibacillus macroides* against toxigenic *Aspergillus flavus* *and Fusarium proliferatum* and Analysis of its mycotoxin minimization potential**

Ahmed L. Mahmoud^1^, Ayat H. Kileny^1*^, Elhagag A. Hassan^1^

^1^Botany and Microbiology Department, Faculty of Science, Assiut University, 71516 Assiut, Egypt

*Corresponding to: Ayat Hassan Kileny, Teaching assistant of Microbiology*

Botany and Microbiology Department, Faculty of Science, Assiut University, 71516 Assiut, Egypt

Tel: 002 01013721523 E-mail: Ayat93@aun.edu.eg

**Supplementary Figure S1**


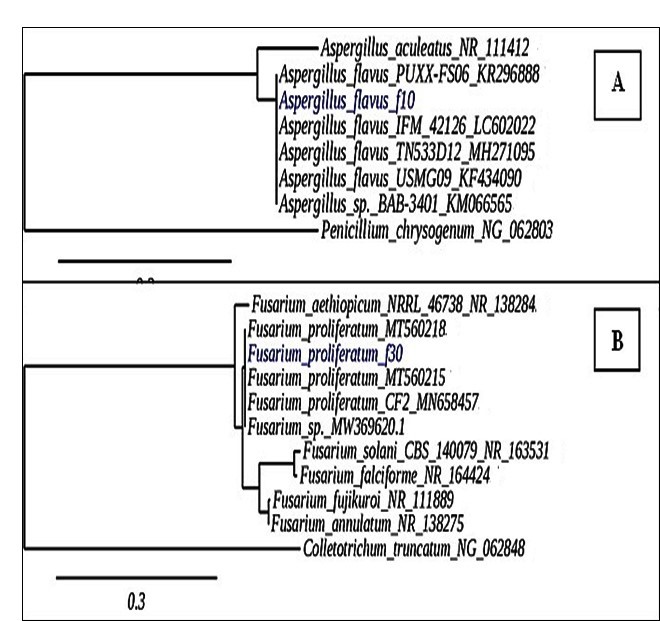
 S1: The phylogenetic trees representing (A) *Aspergillus flavus* f10, (B) *Fusarium proliferatum* f30. Trees were constructed from aligned sequences (ITS) using neighbor-joining methods. Penicillium chrysogenum and Colletotrichum truncatum were used as the out of the group.

**Supplementary Figure S2**

S 2: phylogenetic tree of *Lysinibacillus macroides*. Tree was constructed from aligned sequences (16s rRNA) using neighbor-joining method. Pseudomonas aeruginosa was used as the out of group
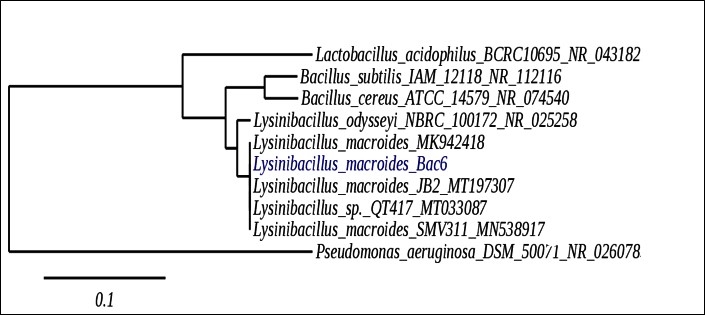
.
